# Inter-observer agreement on varicoceles diagnosis among patients referred to Shiraz Namazi Hospital

**Published:** 2018-10

**Authors:** Saeed Shakeri, Leila Malekmakan, Farhad Manaheji, Taraneh Tadayon

**Affiliations:** 1 *Shiraz Nephro-Urology Research Center, Department of Urology, Shiraz University of Medical Sciences, Zand Avenue, Shiraz, Iran.*; 2 *Shiraz Nephro-Urology Research Center, Shiraz University of Medical Sciences, Khalili Street, Shiraz, Iran.*

**Keywords:** Varicocele, Physical exam, Interobserver agreement

## Abstract

**Background:**

Varicocele is the collection of dilated veins of pampiniform plexus which is the most common cause of infertility occurred in 35-40% of infertile men. Despite all the diagnostic tools exist for varicocele diagnosis; still, a physical examination is the first step.

**Objective:**

This study was done to determine the inter-observer agreement on varicoceles diagnosis.

**Materials and Methods:**

In this cross-sectional study, two expert examiners evaluated 93 men with infertility, scrotal pain, and enlarged scrotum for the presence of varicocele. The urologists examined patients independently in two separate rooms. The inter-observer agreement on varicocele grading analyzed by the Spearman Rho correlation coefficient.

**Results:**

The grading of varicocele were similar in 34 (36.5%) and 59 (63.4%) cases in the left and right side, respectively with correlation coefficient of 0.65 (0.51-0.75) and 0.25 (0.04-0.43), respectively. It implies that inter-observer agreement was substantial for left varicocele assessment and moderate for right varicocele assessment.

**Conclusion:**

Physical exam is the essential diagnostic tool for varicocele. As long as the observers educated with the same standard method the disagreements reduced to the negligible extent.

## Introduction

Varicocele is an abnormal venous dilatation in the pampiniform plexus that affects about 15% of men. It can present with scrotal pain and swelling (1). Approximately 40% of marital infertility is due to the male factor. Varicocele is the most common correctable cause of male infertility, which found in 35% of men with primary infertility and in 80% with secondary infertility )^2^(. Varicocele is a common pathology of the testes characterized by a varicosity of the pampiniform venous plexus of the spermatic cord, with an average incidence of 14.7% )^3^(. Varicocele is often classified as clinical or subclinical. Clinical varicocele is diagnosed and graded based on physical examination )^4^, ^5^(. 

Physical examination is subjective and depends on the experience of the examiners, the body habits of the patient, and the contractile state of the scrotum. Some previous studies reported the low accuracy of physical examination )^4^, ^5^(. Several studies emphasize the difficulty in diagnosis and significant inter-observer variability in varicocele examination even when experienced urologists perform the physical examination (6-13). On the other hand, some other study still mentioned the physical examination as the most important diagnostic tool (14). Although inter-observer variability in the evaluation of varicoceles is a commonly known pitfall of physical examination, it is believed that the clinical evaluation of a varicocele depends on the skill of the surgeon. 

This study aims to evaluate the inter-observer variability in varicocele examination.

## Materials and methods

**Population and study design**

This is a cross-sectional study conducted between January and August of 2014 at Saadi Hospital, Shiraz, Iran. In this present study, we included 99 men presented with infertility, scrotal pain, and enlarged scrotum. Then they were examined for the presence of varicoceles. Patients with a congenital abnormality, previous urogenital surgery, and history of trauma to scrotum were excluded. 

**Varicocele evaluation**

The clinical diagnosis was established on the basis of physical and andrologic examinations with an assessment of varicocele severity on the Dubin-Amelar 1 to 3 scale; Grade 1 varicoceles are small and detectable only with the Valsalva manoeuver. Grade 2 varicoceles are moderate in size and are palpable with the patient standing even without Valsalva manoeuvre, but are not visible. Grade 3 varicoceles are large and visible through the scrotal skin, subclinical varicoceles are those that are not palpable on physical examination and require sonographic imaging for diagnosis (12). Subsequently, two expert urologists examined participants to assess the testis varicocele grading. The examination was done independently in two separate warm and comfortable rooms. The evaluation was done after 5 min standing and also during the Valsalva manoeuvre. The results were recorded and later compared. 

**Ethical consideration**

The study has been approved by the Ethics Committee of Shiraz University of Medical Sciences, Shiraz, Iran (code: IR.SUMS.REC. 1396.S769). Before enrolment, all participants received written and verbal information regarding the aims of the current study. And sign the informed consent form.

**Statistical analysis**

Dates were presented by the number and percentage for categorical variables. For data analyzing, we use SPSS software (Statistical Package for the Social Sciences, Version 19.0, SPSS Inc, Chicago, Illinois, USA) and p<0.05 was considered as the statistical significant level. Agreement on the testis varicocele grading due to its multi-stage pattern, was analyzed with Spearman Rho correlation coefficient (r), with the value of -1 means a perfect negative correlation; very poor if r <0.15, poor if 0.15≤ r ≤0.25, moderate if 0.25≤ r ≤0.40, substantial if 0.40≤ r ≤0.75, and excellent if r >0.75.

## Results

This study conducted to evaluate how much inter-observer variability in varicocele evaluation exists. All of the patients attending the infertility clinic with infertility and scrotal pain. After excluding six patients of all 99 cases, 93 patients with the mean age of 31.3 yr attended in our study. Each man was examined by 2 different urologists in separate clinic rooms. Examiner 1 reported 82.7% (77 patients) with left and 35.1% (33 patients) with right varicocele while the second examiner documented 55.91% (52 patients) with left and 31.91% (30 patients) with right varicocele.

The records of varicocele grading were similar in 34 (36.5%)) and 59 (63.4%) of cases in the left and right side, respectively (Table I). The clinicians were agreed on 12 (29.3%) of grade 0 varicocele, 3 (37.5%) of grade 1 varicocele, 8 (33.3%) of grade 2 varicocele and 10 (50%) of grade 3 varicocele in left testes. In right testes they were agreed on 48 (73.8%) participants of varicocele with grade 0, 10 (47.6%) of varicocele with grade 1, and 1 (14.3%) of varicocele with grade 3. 

The inter-observer agreement on varicocele evaluated by the Spearman Rho correlation coefficient that r=0.65 (0.51-0.75) and r=0.25 (0.04-0.43) were derived for left and right side, respectively. It shows that inter-observer agreement was substantial for left varicocele assessment and moderate for right varicocele assessment (Table II).

**Table I:** Findings of varicocele examination by 2 different examiner

**Examiner 2**							
**Examiner 1**	Left varicocele assessment (n= 93)						
	Grading	Grade 0	Grade 1		Grade 2	Grade 3	Total
	Grade 0	12 (29.3)	24 (58.5)		5 (12.2)	0 (0.0)	41 (100.0)
	Grade 1	1 (12.5)	3 (37.5)		4 (50.0)	0 (0.0)	8 (100.0)
	Grade 2	3 (12.5)	9 (37.5)		8 (33.3)	4 (16.7)	24 (100.0)
	Grade 3	0 (0.0)	1 (5.0)		9 (45.0)	10 (50.0)	20 (100.0)
	Right varicocele assessment (n= 94)						
		Grade 0	Grade 1	Grade 3		Total	
	Grade 0	48 (73.8)	17 (26.6)	0 (0.0)		65 (100.0)	
	Grade 1	11 (52.4)	10 (47.6)	0 (0.0)		21 (100.0)	
	Grade 3	3 (42.9)	3 (42.9)	1 (14.3)		7 (100.0)	

Data presented as n (%).

**Table II:** Inter-observer agreement of varicocele assessment

**Assessment**	**Agreement (CI)** *	**Strength**	**Statistic**	**p-value**
Left varicocele	0.65 (0.51-0.75)	Substantial	Spearman Rho	<0.001
Right varicocele	0.25 (0.04-0.43)	Moderate	Spearman Rho	0.019

* : 95%confidence intervals

## Discussion

Varicocele is the most common cause of infertility in men that can be cured by surgery or radiological embolization (14). Despite of the various imaging modalities introduction, still physical examination mentioned as the essential step in varicocele diagnosis and screening (15, 16). This study evaluated the inter-observer variability in varicocele examination, which was substantial for left and had moderate strength for right varicocele assessment. This contrary difference on agreement of right and left varicocele indirectly can show the inaccuracy of physical examination.

In the previous study conducted by Shakeri and colleagues, we assessed the intra-observer discrepancies (17). In the present study we tried to determine the inter-observer disagreements and the examiner dependency of varicocele diagnosis and grading. To obtain this goal two urologists were selected to exam the same patients in the separate room. The results showed a significant agreement in grading of both right and left varicocele and in estimating size of both testes. There was a slight difference in similarity of right and left varicocele grading in a way that the agreement in left side was more significant than in right side. The contrary difference is present for the size estimating of both testes. This may be due to the difference in anatomical position of left and right spermatic veins. 

Unlike our results, previous study conducted by Carlsen and co-workers revealed the significant inter and intra-observer discrepancies in physical examination of testes (16). In the mentioned study 23 men examined by 9 clinicians from 5 different countries and 6 of the clinicians examined the same patients twice. In the repeated examination only 2 examiners’ results were the same as previous examination. 

Another similar study evaluated the scrotal physical exam operated in 37 patients with un-descendent testis which two observers examined the boys. They revealed a complete agreement in just 13.5% of the patients (18). 

In the mentioned studies examiners were from different countries or may have different years of experience and also different degree while in our study the examiners were from the same country and same university with the same years of experience so they have been educated the same way. As a result, they examine with the same standard method. This may explain the difference between studies.

In our previous study, one urologist examine 113 patients twice. The physical exam was different in 16.81% of cases and we concluded many errors in physical exam no matter to the clinicians’ experience and we recommended not to decide based on physical exam alone (17). In all the studies conducted before, and in the present study, observers’ awareness of participating in the study probably has influenced their examinations since they might have examined more carefully.

Some previous studies revealed poor accuracy of physical examination in comparison to thermography and color Doppler ultrasonography especially in diagnosis of subclinical varicocele. They have recommended color Doppler ultrasonography as a screening method (19, 5). On the other hand, some other studies reported significant inter-ultrasonographer disparities (20). Due to controversies in this subject further studies should be prepared.

## Conclusion

In conclusion based on our results, physical examination is the first step and essential diagnostic tool in varicocele diagnosis. To reduce the inter-observers’ disagreement we must standardize and coordinate the method of examination. Also further studies must be done to eliminate the controversies in this subject.
